# Supplementary material for: Impact of Dairy Intake on Plasma F2-IsoProstane Profiles in Overweight Subjects with Hyperinsulinemia: A Randomized Crossover Trial
Source: Nutrients. 2021 Jun 18;13(6):2088. doi: 10.3390/nu13062088 (PMC8235654; doi:10.3390/nu13062088)

Table S1. Correlations between glycemic parameters and F_2-isoprostanes (IsoPs) after adequate-dairy intake.

	FBG	Fasting Insulin	HOMA-IR
5(*RS*)-5-F_{2c}-IsoP	$p = 0.754$ $r = 0.075$	$p = 0.703$ $r = -0.091$	$p = 0.993$ $r = -0.002$
5-*epi*-5-F_{2t}-IsoP	$p = 0.645$ $r = 0.110$	$p = 0.888$ $r = -0.033$	$p = 0.702$ $r = 0.091$
5-F_{2t}-IsoP	$p = 0.463$ $r = 0.174$	$p = 0.839$ $r = 0.044$	$p = 0.415$ $r = 0.193$
8-F_{2t}-IsoP	$p = 0.551$ $r = 0.142$	$p = 0.552$ $r = -0.142$	$p = 0.853$ $r = -0.044$
15-*epi*-15-F_{2t}-IsoP	$p = 0.722$ $r = -0.085$	$p = 0.853$ $r = 0.044$	$p = 0.799$ $r = 0.061$
15-F_{2t}-IsoP	$p = 0.083$ $r = 0.397$	$p = 0.956$ $r = -0.013$	$p = 0.544$ $r = 0.144$
Total F_2-IsoP	$p = 0.647$ $r = 0.109$	$p = 0.871$ $r = -0.039$	$p = 0.761$ $r = 0.073$

Iso, isoprostane; FBG, fasting blood glucose, HOMA-IR, homeostatic model assessment of insulin resistance, Spearman Partial correlation adjusted for age, sex and BMI, *P<0.05

Table S2. Correlations between glycemic parameters and F_2-isoprostanes (IsoPs) after High-dairy intake.

	FBG	Fasting Insulin	HOMA-IR
5(*RS*)-5-F_{2c}-IsoP	$p = 0.590$ $r = -0.121$	$p = 0.805$ $r = 0.056$	$p = 0.734$ $r = 0.077$
5-*epi*-5-F_{2t}-IsoP	$p = 0.161$ $r = -0.309$	$p = 0.930$ $r = -0.020$	$p = 0.968$ $r = -0.009$
5-F_{2t}-IsoP	$p = 0.809$ $r = -0.055$	$p = 0.676$ $r = 0.094$	$p = 0.570$ $r = 0.128$
8-F_{2t}-IsoP	$p = 0.892$ $r = 0.031$	$p = 0.682$ $r = 0.109$	$p = 0.489$ $r = 0.156$
15-*epi*-15-F_{2t}-IsoP	$p = 0.585$ $r = 0.123$	$p = 0.344$ $r = 0.212$	$p = 0.197$ $r = 0.286$
15-F_{2t}-IsoP	$p = 0.645$ $r = 0.104$	$p = 0.823$ $r = 0.051$	$p = 0.397$ $r = 0.190$
Total F_2-IsoP	$p = 0.631$ $r = -0.102$	$p = 0.732$ $r = 0.075$	$p = 0.612$ $r = 0.115$

FBG, fasting blood glucose, HOMA-IR, homeostatic model assessment of insulin resistance, Spearman Partial correlation after adjusted for age, sex and BMI, *P<0.05

Figure S1. Flowchart of the study

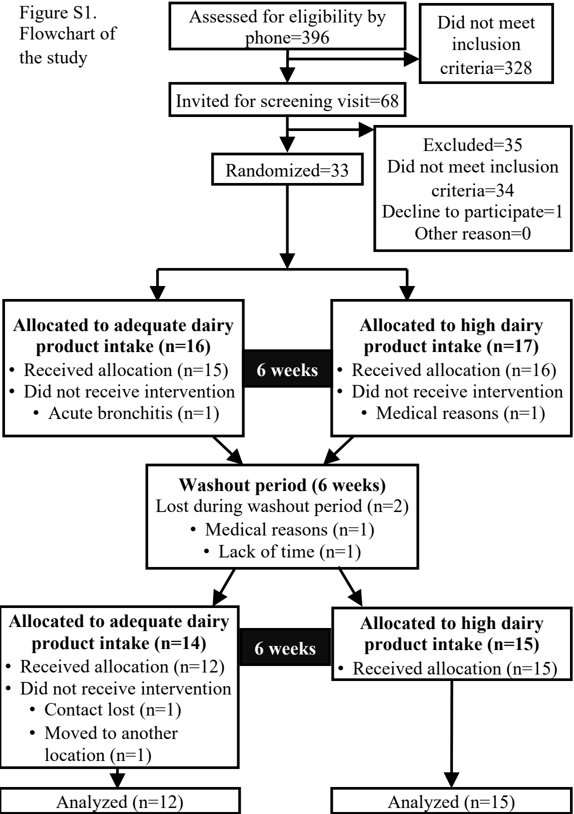

Supplement: Supplementary file 1 [file nutrients-13-02088-s001.zip › nutrients-1206608-supplementary.pdf]
